# The Role of Dafachronic Acid Signaling in Development and Longevity in *Caenorhabditis elegans*: Digging Deeper Using Cutting-Edge Analytical Chemistry

**DOI:** 10.3389/fendo.2016.00012

**Published:** 2016-02-11

**Authors:** Hugo Aguilaniu, Paola Fabrizio, Michael Witting

**Affiliations:** ^1^UMR5262, Ecole Normale Supérieure de Lyon, CNRS, Institut de Génomique Fonctionnelle de Lyon, Université de Lyon Claude Bernard, Lyon, France; ^2^Research Unit Analytical BioGeoChemistry, Department of Environmental Sciences, Helmholtz Zentrum München, Neuherberg, Germany

**Keywords:** aging, steroid signaling pathway, *Caenorhabditis elegans*, analytical chemistry, steroid hormones

## Abstract

Steroid hormones regulate physiological processes in species ranging from plants to humans. A wide range of steroid hormones exist, and their contributions to processes, such as growth, reproduction, development, and aging, is almost always complex. Understanding the biosynthetic pathways that generate steroid hormones and the signaling pathways that mediate their effects is thus of fundamental importance. In this work, we review recent advances in (i) the biological role of steroid hormones in the roundworm *Caenorhabditis elegans* and (ii) the development of novel methods to facilitate the detection and identification of these molecules. Our current understanding of steroid signaling in this simple organism serves to illustrate the challenges we face moving forward. First, it seems clear that we have not yet identified all of the enzymes responsible for steroid biosynthesis and/or degradation. Second, perturbation of steroid signaling affects a wide range of phenotypes, and subtly different steroid molecules can have distinct effects. Finally, steroid hormone levels are critically important, and minute variations in quantity can profoundly impact a phenotype. Thus, it is imperative that we develop innovative analytical tools and combine them with cutting-edge approaches including comprehensive and highly selective liquid chromatography coupled to mass spectrometry based on new methods such as supercritical fluid chromatography coupled to mass spectrometry (SFC-MS) if we are to obtain a better understanding of the biological functions of steroid signaling.

## Introduction

Since its first detailed description by Sydney Brenner in 1974, the nematode *Caenorhabditis elegans* has become a key model organism for biological research (1). Many early studies on fundamental processes, such as apoptosis, development, and aging, were performed using *C. elegans*. The many practical advantages offered by *C. elegans* include fixed and known cell lineage, short development time, and translucent body [reviewed in detail elsewhere (2)]. A normal *C. elegans* life cycle consists of hatching from eggs, four larval stages, L1–L4, and a fertile adult stage, at which point the individual worm is composed of exactly 959 somatic cells and a germline. The hermaphroditic nature of the worm makes it easy to maintain large populations of isogenic animals. The relative simplicity of *C. elegans* and its straightforward life cycle has therefore driven scientists to use this animal model to study various biological processes and uncover important signaling and metabolic pathways.

*Caenorhabditis elegans* possesses a functional steroid signaling pathway that in many ways resembles that found in mammals (3). Although the pathway’s role in development is clear, its impact on longevity is still not well understood. Information gathered to date suggests that the regulation of steroid signaling is complex, and we propose that completely deciphering its function and regulation will require a combination of genetic manipulation of the enzymatic pathways coupled with the development of sophisticated analytical chemistry techniques capable of capturing the molecules. Indeed, many of the small steroid precursor molecules and the genes encoding the key steroidogenic enzymes remain to be formally identified.

Here, we review current knowledge of the components, regulation, and actions of this signaling pathway, as well as the analytical chemical methods that have been employed to date. Finally, we will introduce some promising new tools for the study of steroid signaling, including the use of steroidomics as an untargeted approach to steroid analysis.

## Biological Actions of *C. elegans* Steroids

### Functions during Development

The role of steroid signaling during *C. elegans* development is clearly established. During the larval stages, the worm can sense the environment and make developmental decisions accordingly. When conditions are challenging (food scarcity, high temperature, or crowded environment), young larvae enter into a stress-resistant, non-reproductive Dauer stage (Dauer, German for enduring). Dauer larvae can live for months and retain the capacity to resume development into reproductive adulthood when conditions improve. The steroid signaling pathway is instrumental in this decision-making process. Under favorable conditions, the insulin and TGF-β signaling pathways are activated, resulting in production of the two steroid hormone Δ4- and Δ7-dafachronic acid [often referred to as dafachronic acid (DA)] by the cytochrome P450 DAF-9 (4). DA then binds to the nuclear hormone receptor (NHR) DAF-12 (5) that is no longer repressed by the corepressor DIN-1S/SHARP under these conditions (6). Activation of DAF-12-dependent transcription prevents entry into the Dauer stage and promotes development to reproductive adulthood. When conditions are unfavorable, however, DA is not produced and DAF-12 remains bound to DIN-1S/SHARP, thereby repressing transcription and leading to larval arrest at the Dauer stage. As a result, animals carrying strong *daf-9* mutant alleles are Dauer constitutive while *daf-12* null mutants are Dauer defective. Interestingly, *daf-12* mutants carrying mutations in their ligand-binding domain (LBD) are Dauer constitutive (class 6), in agreement with an instructive role for DAF-12 in the Dauer decision (7). It is important to note that several pathways appear to independently affect the decision to enter into Dauer. For example, *daf-2(e1370)* mutants, which harbor a defective insulin receptor, are Dauer constitutive. However, overexpression of *daf-9* in this strain only partially suppresses their tendency to enter the Dauer stage, and addition of DA induces these animals to arrest at the L3 stage rather than to continue development into gravid adults (5).

Steroid signaling also governs developmental timing. In response to environmental conditions, production of DA and activation of DAF-12-dependent transcription increase the expression of the *let-7* microRNA family (8). These microRNAs are components of the heterochronic pathway, which controls the timing of developmental events in *C. elegans* (9, 10). Mutants defective in *daf-12* or carrying mutations in the microRNAs *mir-48*, *mir-241*, and *mir-84* exhibit disorganized developmental progression, with specific cell divisions being reiterated or skipped (8). The steroid signaling pathway is therefore capable of responding to the environment to control developmental timing through a number of mechanisms. Clearly, this pathway is a central regulator of the *C. elegans* developmental machinery. Since different levels of the steroid hormones may trigger qualitatively distinct signals, analytical methods capable of measuring DA levels with great precision will be necessary to unravel the intricate relationship between steroid production and physiological outcome.

### Functions in Adult Lifespan

Steroid signaling affects the lifespan of adult *C. elegans* through various mechanisms reviewed here below.

#### Temperature-Dependent Modulation of Lifespan

Early experiments showed that *C. elegans* strains incapable of producing DA [i.e., strong *daf-9* mutants (also called class 2 alleles)] were long-lived at low (15°C) but not at warmer (20 or 25°C) temperatures (11–13). This phenotype is not observed in animals carrying weaker *daf-9* alleles, such as *rh50* (class 1 allele), which generally shorten lifespan at 25°C but have no effect at 15 or 20°C. Lifespan extension observed in strong *daf-9* mutants at 15°C is completely dependent on expression of functional DAF-12 (13). Moreover, complementation of *daf-9* mutants with the DAF-12 ligand restores the wild-type lifespan at 15°C, confirming that the long-lived phenotype required DA signaling *via* DAF-12 (12). Interestingly, either addition of DA to mutants solely during development or expression of *daf-9* in the hypodermis or in neuron-like cells (called XXX cells) during development is sufficient for the wild-type lifespan at 15°C to be restored (12). These results suggest that steroid-dependent regulation of lifespan begins during development.

The extended lifespan of *daf-9* null mutants at 15°C is associated with increased stress resistance. Animals carrying either of the *e1406* or *dh6 daf-9* null alleles are resistant to both thermal (35°C) and oxidative (hydrogen peroxide) stress (12), and this resistance is also reversed by addition of DA or introduction of a *daf-12* mutation. Interestingly, animals with low insulin signaling (i.e., mutation of the insulin receptor gene *daf-2*) are also stress resistant; however, in this case, addition of DA is without effect, indicating that the *daf-9* and *daf-2* mutations confer resistance through distinct mechanisms (12). This is consistent with the observation that overexpression of *daf-9* does not affect the long lifespan of *daf-2* mutants (14). However, *daf-9*-dependent stress resistance is partly dependent on DAF-16, the FOXO transcription factor downstream of the insulin signaling pathway. These results show that although longevity and development are both affected by steroid signaling, they involve different regulatory mechanisms.

Several studies have shed light on the involvement of the steroid signaling pathway in temperature-sensitive lifespan regulation. Lee and Kenyon showed that thermosensory defects (ablation of the AFD thermosensory neurons or mutation of *ttx-1* and *ttx-3*, the transcription factors involved in thermosensation) decrease the *C. elegans* lifespan at high temperature (25°C) (15). However, this is not observed in hypomorphic *daf-9(rh50)* mutants or *daf-12* null mutants (15). This finding is in accordance with the observation that steroid signaling is required for lifespan regulation by thermosensory neurons at high temperature. Consistent with this, overexpression of *daf-9* in *ttx-1* mutants restores the wild-type lifespan (15). Finally, recent work has described a role for *daf-41*, the *C. elegans* homolog of p23 cochaperone/prostaglandin E synthase-3, in temperature-dependent modulation of lifespan. The lifespan of these mutants is virtually temperature insensitive since, compared with wild-type animals, their lifespan is longer at 25°C, shorter at 15°C, and no different at 20°C. Interestingly, a *daf-12* null mutation reverses the short lifespan of *daf-41* mutants at 15°C but has no effect at 20 or 25°C (16). The role of steroid signaling in temperature-dependent regulation of lifespan is obviously complex, and much work remains to fully elucidate its involvement.

#### Germline-Mediated Longevity

Steroid signaling controls longevity in germline-less *C. elegans*. In 1999, Hsin and Kenyon showed that laser ablation of the germline precursor cells at the first larval stage extends the lifespan by ~60%, and this extension depends on the somatic gonad, the FOXO transcription factor DAF-16, and the NHR DAF-12 (17). In addition to DAF-12, several other components of the DA biosynthetic pathway are required for lifespan extension though ablation of the germline, including the Rieske-like protein, DAF-36 (18), the cytochrome P450, DAF-9 (12), and the 3-hydroxysteroid dehydrogenase, DHS-16 (18). Although the cytochrome P450 oxidoreductase EMB-8 and the alternative 3-hydroxysteroid dehydrogenase HSD-1 have been identified as enzymes in the steroid signaling pathway, the requirement for germline-dependent lifespan extension of EMB-8 has not yet been tested (19), and HSD-1 is not required (20). These differences of how enzymes involved in the steroid biosynthetic pathway impact the lifespan of germline-less animals may point to the involvement of other steroid hormones, such as the precursors or derivatives of DA. Analytical techniques capable of identifying and quantifying very low levels of steroid hormones will be indispensable for such studies.

Interestingly, animals that lack the entire gonad (germline and somatic gonad) are not long-lived unless they are provided with exogenous DA and possess functional DAF-12 and DAF-16 proteins (21). One possible interpretation for these data is that DA is produced in response to germline ablation, possibly in the somatic gonad itself or in tissues controlled by the somatic gonad. However, direct measurements of DA after germline ablation have produced conflicting results depending on the analytical method used. Compared with wild-type animals, DA levels were either increased (22) or unchanged (23) in *glp-1(e2141ts)* mutants, which are unable to produce a germline. Because *glp-1* mutants are morphologically different from wild-type animals but similar to gonad-less animals, a better approach to resolve this question might be to directly compare DA levels after ablation of the somatic gonad versus the germline. The development of analytical techniques capable of detecting steroid hormones in a tissue-specific manner would be a great advance in the field. In any event, if the lack of DA production in response to germline ablation is confirmed, this might suggest that DAF-12 responsiveness to its ligand is heightened under these conditions (23). How that might occur remains to be determined.

Of note, steroid signaling regulates germline-mediated longevity through molecular strategies similar to those employed during development. For example, *daf-12*-dependent production of the microRNAs *mir-81* and *mir-241* is required for both the heterochronic pathway during development and lifespan extension in response to germline ablation (8, 22). Recently, it was shown that DAF-12 activation in response to germline ablation induces expression of *mir-81* and *mir-241*, which activate DAF-16 by inhibiting the expression of AKT-1 (22). However, the transcriptional activities of the steroid and insulin signaling pathways appeared to be largely independent (24).

#### Lifespan Extension through Dietary Restriction

A recent study used two independent models to show that DA is produced in response to dietary restriction; these were adult wild-type worms subjected to complete bacterial deprivation, and *eat-2* mutants, which have reduced food intake due to a pharyngeal pumping defect (25). Interestingly, two isoforms of DA (Δ4 and Δ7) were augmented by dietary restriction, an observation made possible by the use of carefully refined analytical techniques (26). Although *daf-9* mRNA levels were elevated under these conditions, how DA synthesis is activated by dietary restriction is not clear. Several potential DA biosynthetic pathways exist (27), suggesting that the precise route to DA production can only be resolved if all cholesterol-derived metabolites are measured under the conditions in which DA is produced. Elevated *daf-9* mRNA levels are also observed in *daf-16* and *daf-7* mutants subjected to dietary restriction, suggesting that, in contrast to its regulation during development, steroid signaling in adult *C. elegans* is not controlled by the insulin and TGF-β signaling pathways. Interestingly, activation of the steroid signaling pathway is required for dietary restriction-induced lifespan extension, since weak *daf-9* mutants subjected to dietary restriction do not live longer. Surprisingly, DAF-12 does not appear to be involved in this response; instead, the closely related NHR-8 has been implicated (25). NHR-8 is a master regulator of cholesterol homeostasis in *C. elegans*, but its role in longevity determination is not well understood (28).

In wild-type *C. elegans*, nutrient limitation leads to a large reduction in the size of the germline, but this is not observed in steroid signaling pathway mutants. When germline size reduction is re-established in steroid mutants by mutations in the Notch or TGF-β signaling pathways, which control germline stem cell proliferation by different mechanisms, lifespan extension is also restored (25). Taken together, these results suggest that steroid signaling is capable of affecting the germline, depending on the nutritional status of the animal, and that germline dynamics play a role in dietary restriction-mediated lifespan extension. It is interesting to note that although steroid signaling regulates reproduction in response to nutritional cues in both adult and developing *C. elegans*, the mechanisms involved are profoundly different.

#### Male-Induced Loss of Fitness and Shortening of the Lifespan

*Caenorhabditis elegans* exists predominantly as self-fertilizing hermaphrodites, and males are relatively rare. Under conditions of stress, however, the frequency of males increases markedly. The Murphy and Brunet labs independently reported that mating of hermaphroditic worms with males induces a drastic loss of fitness in the hermaphrodites, characterized by body shrinkage and precocious death (29, 30). Interestingly, Shi and Murphy showed that shrinking and early death are the direct result of mating. DAF-9 and DAF-12 are required for body shrinking and, to a lesser extent, for the rapid decay leading to death, suggesting that shrinking and short lifespan can be uncoupled in this context. They also found that *daf-9* levels in the spermatheca were significantly decreased in response to mating.

**Figure 1:**
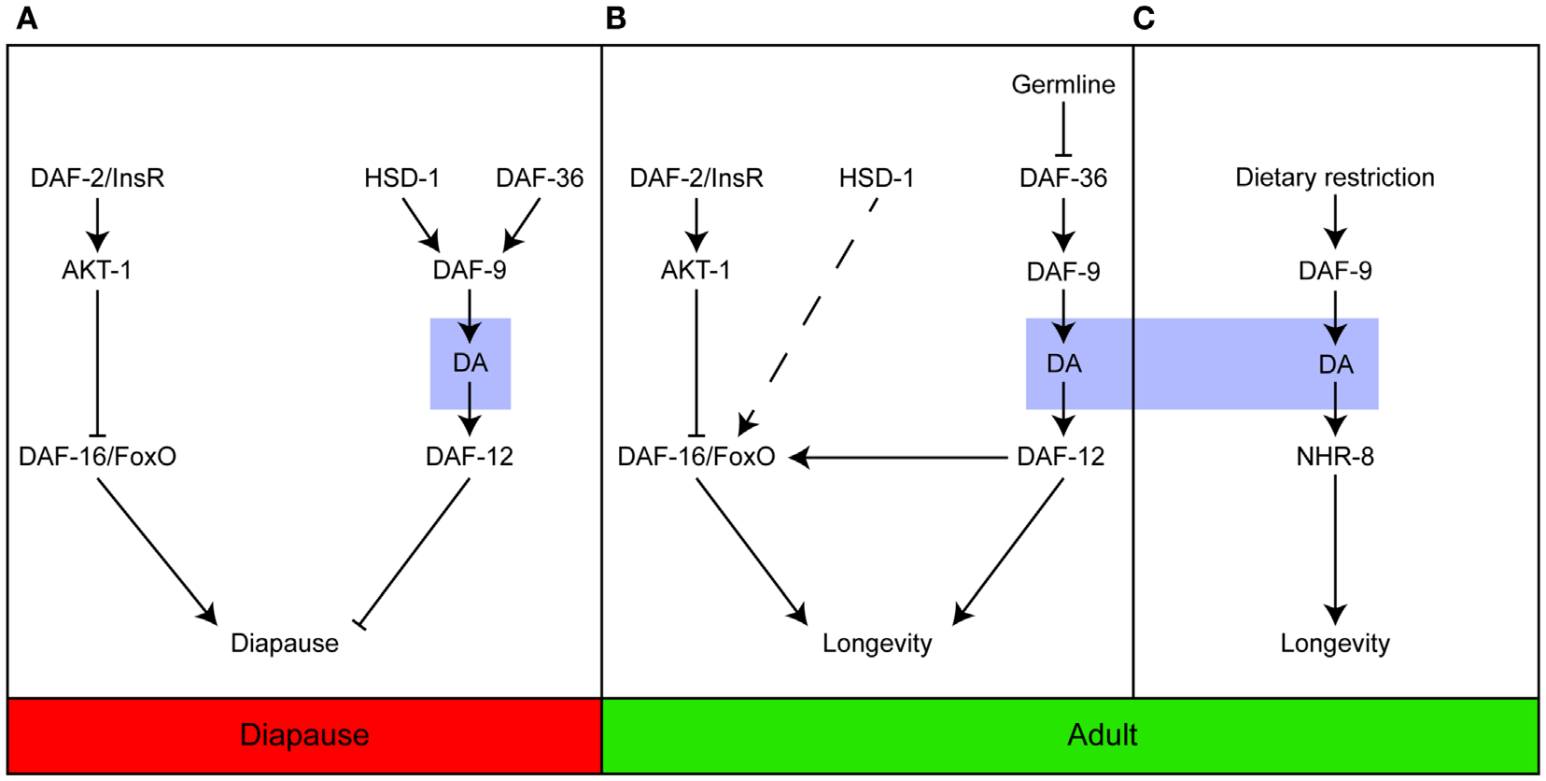
**Dafachronic acid plays important roles in developing and adult worms**. Steroid signaling is important for entry into the Dauer diapause **(A)**; longevity in response to germline ablation **(B)**; and the response to dietary restriction **(C)**. In contrast to **(A,B)**, which are dependent on the activation of DAF-12, dietary restriction induces longevity *via* NHR-8. Adapted from Dumas et al. (31).

Collectively, the studies described here indicate that the steroid signaling pathway plays an important role in controlling the response of developing and adult *C. elegans* to their environments. The pathway regulates the timing of key events that allow the animals to develop and grow and age at a pace in keeping with the challenges imposed by the prevailing conditions, most notably temperature, nutritional availability, and reproductive capacity (Figure 1).

## Current Knowledge of Dafachronic Acid Biosynthesis

Although many enzymes taking part in DA production have been identified, we do not yet know the precise reaction sequence starting with cholesterol. It is important to note that DA actually refers to two molecules, Δ4- and Δ7-DA, which could potentially have different biosynthetic pathways. Motola et al. first identified Δ4- and Δ7-DA as products of the DAF-9-catalyzed reaction and showed that both hormones are direct ligands of the NHR DAF-12 (5). Additional DA biosynthetic enzymes were subsequently identified, as mentioned above, including the Rieske-like oxygenase DAF-36 (18), a *C. elegans* ortholog of 3β-hydroxysteroid dehydrogenase/Δ5-Δ4 isomerase, HSD-1 (19), a 3-hydroxysteroid dehydrogenase, DHS-16, and a NADPH–cytochrome P450 oxidoreductase, EMB-8 (27).

Although the enzyme STRM-1 does not take part in DA biosynthesis directly, it may play important roles in regulating their levels (32). This enzyme adds a methyl group to position 4 in the A ring of DA precursors. As a result, the methylated molecules can no longer serve as substrates of DAF-9, and DAs therefore cannot be produced. Hannich et al. proposed that STRM-1 could regulate Dauer formation by controlling the sterol pool for DA synthesis (32). Notably, although the derived 4-methyl sterols were no longer available for DA biosynthesis, they were sufficient for the nematode molting process (32).

The most recent and comprehensive analysis of enzymes and intermediates in DA biosynthesis was reported in 2014 by Mahanti et al., who used a metabolomics approach (33). This study described several novel forms of DA, including 3-hydroxy derivatives of Δ7-DA, and Δ1,7-DA and Δ0-DA, which have two or no double bonds, respectively. The new molecules were identified after extensive activity-guided fractionation, based on an *in vitro* luciferase assay in HEK293T cells and an *in vivo* Dauer rescue assay, followed by comparison of the metabolomes of *daf-22* and *daf-9 daf-12* animals using nuclear magnetic resonance (NMR) spectroscopy. In this study, *daf-22* mutants were employed as ligand-rich positive controls since the DAF-22 homolog, SCPx, is involved in steroid side chain breakdown, and *daf-9 daf-12* animals were the ligand-deficient negative controls (32). Interestingly, Δ4-DA could not be detected in any samples, suggesting that this isoform may be present at very low concentrations compared with Δ7-DA. *In vitro* and *in vivo* testing of synthetic molecules showed that Δ7- and Δ1,7-DA were very potent DAF-12 ligands, and both were able to rescue *daf-9* phenotypes and restore longevity in *glp-1;daf-9* double mutants. Lastly, the authors examined the effects of mutation of putative biosynthetic enzymes. The results suggested that HSD-1 is involved in the synthesis of Δ7-DA, in contrast to an earlier finding (18). *strm-1* mutants contained higher level of DAs than did wild-type animals, which is also in line with previous results (33).

**Figure 2:**
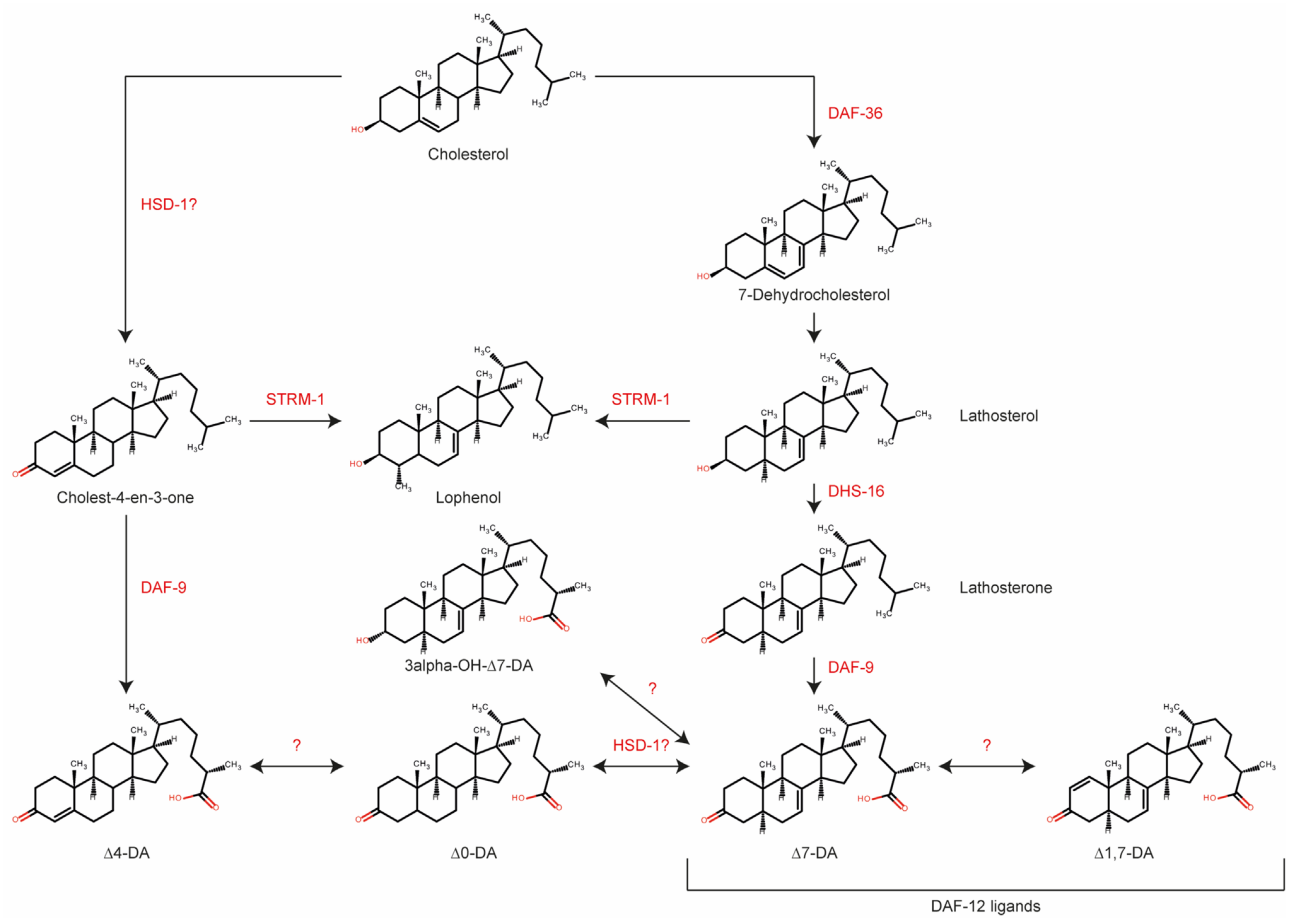
**Biosynthetic pathway for various dafachronic acids based on current knowledge**. Several additional steroids and enzymes may exist. Adapted from Mahanti et al. (33).

Recent results suggest that DA signaling might be more complex than previously anticipated (19, 27, 33). Based on current knowledge, we propose the putative biosynthetic pathway shown in Figure 2. Major gaps in our knowledge still exist; for example, the enzyme converting 7-dehydrocholesterol to lathosterol is not known, although it should theoretically have Δ5 reductase activity. Interestingly, HSD-1 has two paralogs, HSD-2 and -3, but their involvement in DA synthesis has not been rigorously examined to date. Better integration of analytical chemistry and metabolomics tools will facilitate the identification of new enzymes, intermediates, and products in the DA biosynthetic pathway.

Every description of a new gene in the DA biosynthetic pathway suggests the existence of new intermediate products. Table 1 summarizes the DAs and steroid molecules related to DA biosynthesis that, based on current knowledge, could be present in *C. elegans*. The molecules can be roughly divided into three classes: DAs, DA precursors, and other sterols. From a chemical viewpoint, the molecules within a class are very similar, differing only in the position of a double bond or the functional group at position 3, and they have only very small differences in the octanol–water partition coefficient (logP). Furthermore, some molecules in each class have the same exact mass (or both molecules, in the case of “other sterols”). However, even small differences may have significant impacts on the biological effects and potencies of the molecules. Sharma et al. synthesized a number of DA variants with different double bond positions (none, Δ4, Δ5, Δ7) and different stereochemistries at C25 (R or S), and tested their effects in two assays: transactivation of the DAF-12 LBD in HEK293 cells and rescue of the *daf-9* Dauer phenotype (34). They demonstrated that Δ7-DA is the most potent activator and that the double bond position is more important than the stereochemistry at C25 (34). Molecular simulations showed that interaction of the DAF-12 LBD with the 25R isomer is energetically more favorable (35). New molecules are expected to exhibit properties based on the similarity of their chemical structures. Therefore, novel and highly selective and/or efficient separation methods that will facilitate a comprehensive analysis of the *C. elegans* steroidome content are needed.

**Table 1:** **Molecular properties of dafachronic acids and known precursor substances**.

Name	Exact mass	Formula	LogP	ChEBI ID	Class
(25S)-Δ1,7-DA	412.29774	C27H40O3	6.25	CHEBI:83137	DA
(25S)-Δ7-DA	414.31339	C27H42O3	6.26	CHEBI:78699	
3α-OH-(25S)-Δ7-DA	416.32904	C27H44O3	6.05		
(25S)-Δ4-DA	414.31339	C27H42O3	6.62	CHEBI:71560	
(25S)-Δ0-DA	416.32904	C27H44O3	6.66	CHEBI:78701	
3β-OH-(25S)-Δ7-DA	416.32904	C27H44O3	6.05		
Cholesterol	386.35486	C27H46O	7.11	CHEBI:16113	DA precursor
7-Dehydrocholesterol	384.33921	C27H44O	6.71	CHEBI:17759	
Lathosterol	386.354866	C27H46O	7.11	CHEBI:17168	
Lathosterone	384.33921	C27H44O	7.32	CHEBI:71550	
4-Cholesten-3-one	384.33921	C27H44O	7.68	CHEBI:16175	
4α-Methyl-5α-cholest-7-en-3β-ol (Lophenol)	400.37051	C28H48O	7.48	CHEBI:18378	Others
4α-Methyl-5α-cholest-8(14)-en-3β-ol	400.37051	C28H48O	7.43	CHEBI:71581	

*Exact mass is important for mass spectrometric detection*.

*LogP = water/octanol partition coefficient*.

## New Approaches for the Analysis of Known Steroids and Identification of Unknown Steroids in *C. elegans*

### Analytical Approaches for the Analysis of *C. elegans* Steroids

To date, only targeted methods, based mostly on gas chromatography (GC) or liquid chromatography (LC) coupled to mass spectrometry (GC-MS and LC-MS, respectively), have been employed for analysis of *C. elegans* steroids. Direct analysis of substrate and product levels is crucial for assessing enzymatic activities. The analytical techniques for steroids range from simple fractionation using solid phase extraction materials or 2D-thin layer chromatography to highly specific technologies, such as LC-MS/MS or 2D-NMR. Furthermore, chemical synthesis is essential for confirming the identity of newly isolated compounds. GC is the method of choice for molecules that are volatile or can be made volatile by appropriate derivatization, whereas LC is preferable for more polar steroids such as bile acids. Chromatography plays a key role in the analysis of *C. elegans* steroids because several molecules have the same exact mass (Table 1), making separation critical. Because many compounds have similar logP values, the separation methods must also be highly selective.

### Methods for the Quantification of *C. elegans* Steroids

Determining the quantity of different steroids *in vivo* is of paramount importance for understanding regulatory circuits, but the identity of the steroids must be known in order to quantify them. Both GC-MS/MS and LC-MS/MS have been used to analyze steroids in *C. elegans* to substantiate results obtained with genetic tools. Here, we discuss some of these methods.

The method used by Broué et al. for steroid analysis is based on extensive fractionation using solid phase extraction and HPLC, followed by derivatization with heptafluorobutyric acid anhydride and GC-MS/MS analysis. A large quantity of worms (5 × 10^3^ to 3 × 10^5^) are required for this procedure but it has good sensitivity (lower fg) (36). However, the authors did not measure either DAs or their precursors.

Motola et al. first identified the DAs using a single quadrupole LC-MS system for chemical analysis. Separation was achieved by a C18 column with a methanol (MeOH) to MeOH/acetonitrile (ACN)/water gradient and selected ion monitoring (SIM) analysis of different masses corresponding to 4-cholesten-3-one, 1,4 cholesten-3-one, and 3-keto-4-cholestenoic acid in positive mode and 3-keto-4 cholestenoic acid in negative mode (5).

Wollam et al. used a combination of LC-MS/MS and GC-MS/MS to analyze their *C. elegans* samples. For LC-MS/MS, a triple quadrupole instrument was employed to follow levels of lathosterone, lathosterol, 7-dehydrocholesterol, and 4-cholesten-3-one. Separation was achieved by a C18 column with a 90–100% MeOH gradient. DA levels were also analyzed using GC-MS/MS (27). Overall, GC-MS appears to be the preferred method for analysis of *C. elegans* steroids since it was also employed by Shen et al. (22) and Mahanti et al. (33). For this technique, samples must be derivatized to increase their volatility. Shen et al. used multiple reaction monitoring on a GC-MS/MS system for selective detection. Multiple reaction monitoring is based on the selection of a mass of interest, followed by fragmentation of the molecule in a collision cell, and detection of specific fragments to be obtained and quantified. This is usually performed with a triple quadrupole or quadrupole time of flight (Q-ToF) instrument.

Using SIM, Mahanti et al. were able to follow production of Δ7-DA, Δ1,7-DA, Δ0-DA, Δ4-DA, 3α- and 3β-OH-Δ7DA, and 4-cholesten-3-one (33). Quantification of the different DAs was performed using response factors determined from synthetic standards, and the MS response was linear between 10 pg and 5 ng. Interestingly, these authors were unable to detect Δ4-DA in their samples, although 4-cholesten 3-one was detected in amounts similar to the Δ7-DA precursor lathosterone (33).

Liquid chromatography coupled to mass spectrometry is an interesting alternative to GC-MS because it does not require prior derivatization. In particular, bile acids such as DAs are amenable to electrospray ionization (ESI) due to the carboxylic acid functionality. ESI is used to ionize molecules in the LC effluent. Li et al. described an even more sensitive method for quantification of certain DAs, in which samples were derivatized with 2-picolylamine to further decrease the detection limit (37). The authors evaluated the method with respect to recovery after extraction with chloroform/MeOH and various quantification methods for their precision, accuracy, and limit of detection (LOD). The best on-column LOD (1 pg) was obtained with high-resolution SIM. As proof-of-principle, Li et al. applied their method to detect DAs at different developmental stages of wild-type, *daf-9, daf-12*, and *daf-36* animals. DAs were undetectable in the double mutant compared with wild-type animals, and levels in the *daf-36* mutants were intermediate between the two. DA levels were also measured in two different *daf-2* alleles, *daf-7*, and *daf-11* mutants (37). Although the method is very sensitive, its major drawback is that it cannot distinguish between the Δ4- and Δ7-DA isomers.

Sardella et al. used a click quinidine-based chiral stationary phase to separate C25 epimers of DAs (38). Retention was based on anion exchange, whereas separation of stereoisomers was based on stereoselective H-bonding. Detection was carried out at 220 nm. Although the method was qualitative and only tested with chemical standards, the eluent they used is MS compatible and the method could potentially be used for quantification in biological samples. However, this remains to be determined (38).

We recently published a method for the separation of Δ4- and Δ7-DAs using a novel C18 sub-2 μm core–shell particle column in combination with an acetonitrile (ACN) gradient, which allowed independent quantification of the two isomers. We tested a number of gradients for separation of the two isomers and found that a focused gradient with a 65% isocratic step was the best solution. The method was evaluated for extraction efficiency and LOD (26). In contrast to Li et al., we used 100% MeOH for extraction but obtained similar extraction recoveries. Although our LOD was slightly higher than that of Li et al. at ~5 pg per isomer, the major advantage of our method is separation of the isomers. Moreover, we successfully applied this method to quantify changes in both DA isomers in animals subjected to bacterial deprivation (25, 26).

GC-MS and LC-MS each have advantages and disadvantages. Although GC-MS enables high-resolution separation with chromatographic peak widths of several seconds, its drawbacks include derivatization is necessary to increase volatility, separations are carried out at temperatures exceeding 200°C, and columns show only restricted selectivities. In addition, to achieve good separation long runtimes >45 min are needed. GC-MS has been used to analyze steroids for decades and is a well-established method readily available to many labs. By contrast, LC-MS offers a wide variety of column materials with various selectivities for the separation of different, structurally closely related, isomers. No derivatization is needed, but it can be used to increase the sensitivity for weakly ionizing molecules. However, despite increasingly widespread use, LC-MS is not accessible to all labs and dedicated methods must be developed for demanding separations, as is the case for Δ4 and Δ7-DA. Compared to GC-MS, LC-MS run times are generally shorter with around 30–40 min.

### New Analytical Approaches for the Discovery and Analysis of *C. elegans* Steroids

Numerous methods have been described for targeted analysis of known *C. elegans* steroids. However, there clearly exists a need not only for improved, faster, and more sensitive methods but also for methods to identify new and potentially bioactive intermediates or products. Non-targeted metabolomics – the systematic analysis of all small molecules in a system or organism – therefore holds great promise for the discovery of novel steroid metabolites and conjugates. Mahanti et al. have already provided remarkable proof-of-concept for this approach using NMR (33). The major drawback of NMR is its low sensitivity compared to MS, and the large sample quantities required can be a serious limitation for large-scale analysis. Non-targeted MS-based metabolomics approaches have been used in *C. elegans* research for a variety of purposes; for example, metabolic profiling of *daf-2* mutants (39) and the development of a comprehensive method for analysis of lipid content (40). Direct infusion ion cyclotron resonance Fourier transform mass spectrometry (DI-ICR-FT/MS), which has very low sample consumption and ultra-high resolution and sensitivity, is a novel tool for analysis of small molecules and holds great promise for non-targeted metabolomics in *C. elegans* (41). In conjunction with sophisticated data analysis tools, this technology enables identification of novel metabolites in small samples (42, 43). Despite its sensitivity, DI-ICR-FT/MS does have some limitations since it cannot discriminate between isomers with the same molecular formula and exact mass because it does not employ chromatographic separation.

Steroidomics, the non-targeted analysis of steroids, is a sub-category of metabolomics or lipidomics. Similar to the other “omics,” its goal is to comprehensively detect and quantify all steroids in a cell or even an organism. LC-MS using reversed-phase separation is currently the most employed technique (42, 43), but other methods will eventually have to be developed to accommodate the growing complexity and number of isomeric structures to be identified and quantified. Similar to non-targeted metabolomics and lipidomics, steroidomics employs generic separation methods to allow detection of the maximal number of metabolites. However, generic methods could fail to separate isomers exhibiting subtle structural differences. A reversed-phase method based on a water–ACN gradient with formic acid additive is routinely used in our lab for metabolic profiling, and although it can detect DAs, it cannot separate the Δ4 and Δ7 isomers (our unpublished work). Thus, novel approaches, such as improved stationary phases, are required to facilitate separation of isomers during profiling.

A promising tool for future steroidomics applications is supercritical fluid chromatography (SFC), which is currently experiencing a revival. Although this technique has been known for decades, it has been largely overshadowed by HPLC and GC. However, SFC combines the best features of both methodologies: the different column selectivities of HPLC and the efficiency of GC. SFC uses supercritical CO_2_ as the eluent, which has low viscosity and high diffusivity. Furthermore, supercritical CO_2_ is miscible with a wide range of solvents, including MeOH and iProH, and a number of additives (e.g., ammonium hydroxide and ammonium acetate) can be used to fine-tune the separation, peak shapes, and sensitivity. In 2013, Taguchi et al. reported a supercritical fluid chromatography coupled to mass spectrometry (SFC-MS)-based method for the analysis of conjugated and unconjugated bile acids using a sub-2 μm amide column (44). They detected a total of 25 bile acids in rat plasma that was directly injected without extraction or further sample preparation (44). Similarly, SFC coupled to Q-ToF–MS was previously used for non-targeted analysis of rat and dog bile (45). Here, this technique was established as a viable alternative for metabolic profiling. Compared to GC- and LC-MS, SFC-MS yields short runtimes below 15 min. Therefore, SFC-MS holds great promise for use in *C. elegans* research as a superior method for resolving structurally closely related steroids.

These non-targeted analysis techniques allow detecting a wider range of molecules, including some that may not be detectable by targeted or focused analysis. These methods can also lead to the identification of yet unknown molecules (not described in any database), although this is a tedious and often time-consuming task. It requires expert analysis of tandem MS or 1D- or 2D-NMR-spectra, purification or synthesis of a reference standard, and testing of biological activity. One example is the discovery of novel dafachronic acid species by Mahanti et al. (33). Total time until final verification and biological testing took over 9 months (Franck Schroeder, personal communication). In addition, these techniques usually require specialized equipment and data analysis strategies. This is especially true for SFC-MS, not as widespread as GC- or LC-MS, where special pumps, auto sampler, and backpressure regulators are employed. Additionally, the separation mechanism can be different from LC or GC leading to counterintuitive separation results, which has be analyzed and evaluated carefully by a specialist.

Genetic evidence strongly suggests that many steroidogenic enzymes have yet to be identified and characterized. Furthermore, genetic studies have also raised the possibility that steroid synthesis pathways may operate in a tissue-specific manner. *C. elegans* XXX cells have been hypothesized to be a potential site for DA production, but DAF-9, the enzyme responsible for DA production, is also found in the somatic gonad, spermatheca, and hypoderma. Tissue-specific analysis of steroids is therefore required to address this. One potential method is imaging MS; for example, using matrix-assisted laser desorption ionization (MALDI) in combination with high resolving mass spectrometers, such ICR-FT/MS (46). Although imaging of steroid compounds has already been described using MALDI-ICR-FT/MS (47), current commercially available instruments are unable to produce a detailed analysis of the worm’s morphology with satisfactory resolution. Several proof-of-principle papers have been described (46–49), but they had limited resolution with no mean to finely analyze metabolites levels within the animals. In this regard, two obvious methodological advances needed are new sample preparation methods to enable visualization of metabolites within the worm and its tissues, and novel detection instruments aimed at smaller spatial resolutions of <5 μm (50, 51).

New analytical techniques hold great promise to identify new steroid signaling molecules, better understand biosynthesis, and enable precise quantification of more molecules. However, most recent tools, such as SFC, are available to only a few laboratories specialized in this field. In order to truly democratize such technique, an effort will be necessary in the future to make it available to more laboratories.

## Conclusion

Although steroid hormones and their biological roles have been studied for over a century, we do not yet have a complete picture of their synthesis or function. With its central role during development and in adulthood, the steroid signaling pathway is of great interest for understanding complex biological traits such as aging. *C. elegans* is an excellent model organism for the development of new techniques that should significantly improve our ability to study steroid signaling and its biological roles. Recent publications show that DA, the worm-specific bile acid, can exist as various isoforms, suggesting that it has more complex functions than anticipated. Furthermore, there are undoubtedly enzymes, substrates, and products still to be identified. Genetics and analytical chemistry are extremely powerful tools individually, but in combination they enable an approach to identify and characterize the functions of steroid molecules that is currently beyond reach. Steroid signaling often relies on subtle perturbations in the levels of specific molecules, and current detection methods do not have the sensitivity to record such variations. Novel approaches, such as steroidomics and SFC-based analyses, will therefore take us nearer to our goal of obtaining a complete picture of the diverse biological roles of steroids.

## Author Contributions

HA, PF, and MW wrote the manuscript. MW was more involved in the analytical chemistry part while HA and PF were in charge of the biological part.

## Conflict of Interest Statement

The authors declare that the research was conducted in the absence of any commercial or financial relationships that could be construed as a potential conflict of interest.
